# Revision of the Tribe Xyelini (Hymenoptera: Xyelidae) and New Fossil Records from the Lower Cretaceous of Liaoning Province, China †

**DOI:** 10.3390/insects16121253

**Published:** 2025-12-10

**Authors:** Xiaoqin Li, Alexandr P. Rasnitsyn, Jialiang Zhuang

**Affiliations:** 1 College of Life Sciences, Capital Normal University, 105 Xisanhuanbeilu, Haidian District, Beijing 100048, China; lxqnzbxxzj@163.com; 2 Paleontological Institute, Russian Academy of Sciences, 123 Profsoyuznaya ul., 117647 Moscow, Russia; alex.rasnitsyn@gmail.com; 3 Department of Palaeontology, Natural History Museum, Cromwell Road, London SW7 5BD, UK; 4 Key Laboratory of Biodiversity Conservation of National Forestry and Grassland Administration, Ecology and Nature Conservation Institute, Chinese Academy of Forestry, Beijing 100091, China

**Keywords:** Xyelidae, Xyelini, sawfly, Huangbanjigou, Lower Cretaceous

## Abstract

Three new species—*Enneoxyela aculeata* sp. nov., *Enneoxyela eucalla* sp. nov., and *Hemixyela elongata* gen. et sp. nov.—are described and illustrated from the Lower Cretaceous Yixian Formation at Huangbanjigou, Liaoning Province, China. Meanwhile, two new genera are established by the species previously described after re-examination, *Tugnuxyela* gen. nov. based on *T. tugnuica* comb. nov. (Rasnitsyn, 1982), from the Lower Jurassic of Transbaikalian in Russia, and *Junfengixyela* gen. nov. based on *J. cenozoica* comb. nov. (Zhang, 1989) from the Miocene of Shandong in E. China. This is the first occurrence of the tribe Xyelini in the Cretaceous of China. A male specimen is described for the first time in Mesozoic Xyelini; it is found sufficiently similar to the known (Cenozoic) males of the tribe, including the genitalia twisted for 180°. Keys to the genera of Xyelini and to the species of *Enneoxyela* are provided.

## Simple Summary

One new genus and three new species are described from fossil specimens found in Northeastern China. This represents the first record of the tribe Xyelini in the Cretaceous of China. A male specimen is described for the first time in Mesozoic Xyelini with its 180° twisted genitalia. Additionally, two new genera are established, each based on previously described species. In this work, the keys to the genera of Xyelini and to the species of *Enneoxyela* and an updated catalog of fossil Xyelini are provided.

## 1. Introduction

The family Xyelidae Newman, 1834 belongs to the Hymenopteran suborder-grade Symphyta. Both morphological and molecular studies consistently place the xyelids at the basal-most lineage within Hymenoptera [1,2,3,4,5]. Therefore, investigating the diversity and evolutionary history of Xyelidae provides a key window into the early diversification of Hymenoptera. The oldest known fossil records date back to the Middle Triassic. Xyelidae flourished during the Mesozoic, reached peak diversity in the Middle–Late Jurassic and the Early Cretaceous [6,7]. Currently, the Xyelidae comprise five of the following subfamilies: Archexyelinae Rasnitsyn, 1964; Macroxyelinae Ashmead, 1898; Xyelinae Newman, 1834; Madygellinae Rasnitsyn, 1969, and Daohugoinae Rasnitsyn and Zhang, 2004 [1,6,8,9], with >150 fossil and 63 extant species (Paleobiology Database, https://paleobiodb.org/) as of 20 November 2025 [10].

The subfamily Xyelinae is composed of two of the following tribes: Xyelini Newman, 1834, and Liadoxyelini Rasnitsyn, 1966. The former differs from the latter by its forewing vein 1-Rs longer than 1-M. To date, reported Xyelini fossil records comprise five genera and 19 species, from the Middle Jurassic to the Cenozoic [11]. There are two extant genera with 52 species, most of which belong to the genus *Xyela* Dalman, 1819 [12,13]. Mesozoic fossils have been reported from Russia and Kazakhstan, totaling 12 species belonging to five of the following genera: *Yanoxyela* Ren et al., 1995, *Eoxyela* Rasnitsyn, 1965, *Xyelisca* Rasnitsyn, 1969, *Enneoxyela* Rasnitsyn, 1966, *Spathoxyela* Rasnitsyn, 1969 [11]. All specimens were reported in the 20th century and often based on brief descriptions. The most recent comprehensive review and identification of Cenozoic taxa within this tribe was conducted by Rasnitsyn in 1995. Although Zheng et al. [14] recently compiled a key for the family Xyelidae and provided some key characteristics for Xyelini, no further comprehensive taxonomic revision of the tribe has been conducted to date. Therefore, it is necessary to systematically re-examine the taxa within this tribe, particularly the Mesozoic representatives.

We discovered one new genus and three new species in the Yixian Formation at Huangbanjigou, enriching the species record of Cretaceous Xyelidae in China, particularly the diversity of the tribe Xyelini. We also re-examined the taxa of the tribe and described two new genera based on previously described species from the Lower Jurassic of Russia and Miocene of China, originally described in the genera *Eoxyela* and *Xyela*, respectively. Additionally, we provide the keys to genera of the tribe Xyelini and to the species of the genus *Enneoxyela*. The results presented herein give a new understanding of the diversity of Xyelidae in the Mesozoic.

## 2. Materials and Methods

All the specimens were collected from the Lower Cretaceous, Yixian Formation, at Huangbanjigou, Chaomidian Village, Shangyuan Township, Beipiao City, Liaoning Province, China (41°6′ N, 120°8′ E). They are housed in the Key Lab of Insect Evolution and Environmental Changes, the College of Life Sciences and Academy for Multidisciplinary Studies, Capital Normal University (CNUB; Dong Ren, curator), in Beijing, China.

All specimens were imaged using a Nikon SMZ25 stereomicroscope equipped with a Nikon DS-Ri2 digital camera and automatic Z-axis stacking software. Measurements were taken using Adobe Photoshop 2021 (Ver. 22.3.0). Line drawings were prepared using PaintTool SAI Ver. 2; figures assembled using Adobe Illustrator 2020 (vers.24.1.0).

The forewing venation nomenclature follows Zheng et al. [14]. Rs1 and Rs2, respectively, represent the Rs branch closest to the pterostigma and the branch immediately following it.

Diagenetic matrix distortion, which can deform the enclosed fossils and undermine measurement accuracy, is a known complication in the study of fossil insects such as Xyelidae [15]. Fortunately, insectiferous deposits of the Yixian Fm. demonstrate no deformations of this sort sufficient to compromise our measurements to an appreciable extent.

## 3. Results

Systematic paleontology

Order Hymenoptera Linnaeus, 1758

Superfamily Xyeloidea Newman, 1834

Family Xyelidae Newman, 1834

Subfamily Xyelinae Newman 1834

Tribe Xyelini Newman, 1834

Type genus. *Xyela* Dalman, 1819.

Diagnosis. Unlike Liadoxyelini Rasnitsyn, 1966, another tribe of Xyelinae, Xyelini, having 1-Rs longer than 1-M, and 1m-cu not shorter than 0.7 of 3-Cu.

Genera included.

Type genus with three of the following subgenera: *Xyela* s. str. with ca. 50 extant and five extinct mid-Cenozoic species in Eurasia and N. America, *X.* (*Pinicolites* Meunier, 1920) with one living species in NW USA and one extinct one in Oligocene of Germany, and *X.* (*Mesoxyela* Rasnitsyn, 1965) with one species in Early Cretaceous of Transbaikalian in Russia;

*Eoxyela* Rasnitsyn, 1965, with one extinct species in the Late Jurassic of Kazakhstan;

*Enneoxyela* Rasnitsyn, 1966, with nine extinct species in the Late Jurassic of Kazakhstan, Early Cretaceous of Transbaikalian in Russia, and of NE China;

*Junfengixyela* gen. nov., with one species in the Miocene of China;

*Pleroneura* Konow, 1897, with 12 living species in Eurasia and N. America;

*Spathoxyela* Rasnitsyn, 1969, with one species in the Early Cretaceous of Transbaikalia, Russia;

*Tugnuxyela* gen. nov. with one species in the Early Jurassic of Transbaikalian in Russia;

*Yanoxyela* Ren et al., 1995, with one species in the Middle Jurassic of Hebei in China;

*Xyelisca* Rasnitsyn, 1969, with one species in the Early Jurassic of Transbaikalian in Russia.

Remarks. Since the last revision of the tribe [16], a considerable amount of new information has accumulated that, along with the data presented herein, makes it necessary to re-arrange the system of Xyelini once again. The results of the revision are summarized in the key to genera below.

**Key to genera and subgenera of the tribe Xyelini**

  1. R sinuate before the Rs base. Cell 1mcu less than twice as long as wide (except for *Xyelisca* and *Mesoxyela*). ······················································································· 2

  - R straight before the Rs base. Cell 1mcu two times or more as long as wide. ····· 6

  2. Rs+M longer than 1-Rs. Cell 1mcu more than twice as long as wide. Flagellomere 1 and legs very narrow. ·············································· *Xyelisca* Rasnitsyn, 1969

  - Rs+M much shorter than 1-Rs. Cell 1mcu less than twice as long as wide (except for *Mesoxyela*). Flagellomere 1 and legs not very narrow. ···································· 3

  3. Sc ending at both C and R near each other and well before the Rs base. Ovipositor upcurved, with sheath gradually narrowing to an acute apex. ······························································································································· *Pleroneura* Konow, 1897

  - Sc ending at C distant from that at R, or Sc trunk approaching R, and their connection point indistinct. Ovipositor variable but never as above. ······················· 4

  4. Sc trunk closely approaching R, and their connection point indistinct. ······························································································· *Xyela* Dalman, 1819 s. str.

  - Sc trunk distant from R, Sc_2_ distinct. ········································································ 5

  5. Cell 1mcu less than twice as long as wide. Rs+M very short or absent (replaced with 1r-m) ······························································· *Xyela* (*Pinicolites* Meunier, 1920)

  - Cell 1mcu more than twice as long as wide. Rs+M longer than 1-M. ·································································································· *Xyela* (*Mesoxyela* Rasnitsyn, 1965)

  6. Rs+M about as long as 1-M. ······················································································ 7

  - Rs+M about twice as long as 1-M. ·········································································· 10

  7. 2r-rs well behind the mid-length of the pterostigma. Apex of Rs1 closer to Rs2 than to pterostigma. Ovipositor straight, saw-like (not needle-like, narrow), longer than half of forewing length. ··························· *Spathoxyela* Rasnitsyn, 1969

  - 2r-rs near midlength of the pterostigma. Apex of Rs1 closer to pterostigma than to Rs2. ··························································································································· 8

  8. Sc branches ending at C and R at about the same level before the Rs base. Ovipositor short, downcurved. ····················································· *Tugnuxyela* gen. nov.

  - Sc branches ending at C distal of, and at R distinctly basal of, Rs base. ············· 9

  9. Cell 1mcu wide: 1m-cu and 2-Cu distinctly longer than 1-M. Flagellomere 1 not longer than half the head width. ········································· *Junfengixyela* gen. nov.

  - Cell 1mcu narrow: 1m-cu and 2-Cu about as long as 1-M. Flagellomere 1 longer than half the head width. ········································· *Yanoxyela* Ren et al., 1995

  10. Ovipositor short, downcurved, exserting for about the length of the pterostigma ··································································································· *Eoxyela* Rasnitsyn, 1965

  - Ovipositor straight, exserting for much longer than the pterostigma length. ······ 11

  11. Ovipositor exserting for more than half of the forewing length. ···································································································································· *Hemixyela* gen. nov.

  - Ovipositor exserting for less than half of the forewing length.··························································································································· *Enneoxyela* Rasnitsyn, 1966

Genus *Enneoxyela* Rasnitsyn, 1966

*Enneoxyela* Rasnitsyn, 1966, p. 73

Type species. *Enneoxyela crassicauda* Rasnitsyn, 1966; Late Jurassic of Kazakhstan.

Diagnosis. Forewing with R straight before Rs base. 2r-rs before or slightly behind the midlength of the pterostigma. Rs1 apex only exceptionally closer to Rs2 than to pterostigma. Rs+M about twice, or more than twice, as long as 1-M. Ovipositor flat, saw-like (not needle-like, narrow), straight, with sheaths (exserted part of ovipositor) shorter than half forewing length.

Species included. *E. atra* (Rasnitsyn, 1966), *E. compressicauda* Rasnitsyn, 1966, *E. crassicauda* Rasnitsyn, 1966, *E. karatavica* (Rasnitsyn, 1965), and *E. punctata* (Rasnitsyn, 1965) from the Late Jurassic of Kazakhstan; *E. piniciola* (Rasnitsyn in Krassilov, Rasnitsyn, 1982, comb. nov.) from the Early Cretaceous of Transbaikalian in Russia; *E. aculeata* sp. nov. and *E. eucalla* sp. nov. from the Early Cretaceous of NE China. *E.? sibirica* Rasnitsyn, 1969, from the Early Cretaceous of Transbaikalian in Russia, might belong here as well, which needs examination of conspecific fossils with an ovipositor to confirm.

Remarks. The genus diagnosis is refined herein, resulting in *Spathoxyela pinicola* Rasnitsyn in Krassilov, Rasnitsyn, 1982, fitting into this genus rather than into *Spathoxyela* Rasnitsyn, 1969, because of the forewing Rs+M long, 2r-rs is more distant from the apex of the pterostigma, and the ovipositor is shorter than half the length of the forewing. In contrast, *Xyela cenozoica* Zhang, 1989, which was transferred tentatively to *Enneoxyela* by Rasnitsyn [16], is further moved to *Junfengixyela* gen. nov. herein. For more detailed discrimination of genera in the tribe Xyelini, see the key above.

**Key to the species of *Enneoxyela***

  1. Body length without ovipositor 12 mm. Flagellomere 1 half as long as head wide Ovipositor short, narrow. ····················································· *E. atra* (Rasnitsyn, 1966)

  - Body less than 10 mm long. ······················································································ 2

  2. Flagellomere 1 as long as the head is wide. Ovipositor flat, narrowing only distally. Forewing 5.5 mm long. ······················································ *E. aculeata* sp. nov.

  - Flagellomere 1 distinctly shorter than the head width. ········································· 3

  3. Ovipositor sheath 2.7–3 mm long, more than 0.4× as long as forewing. ············· 4

  - Ovipositor sheath 2.2 mm long or less. ··································································· 5

  4. Ovipositor flat, sheath 3 mm long. ···················· *E. compressicauda* Rasnitsyn, 1966

  - Ovipositor thick, needle-like, sheath 2.7 mm long. ·········································································································································· *E. crassicauda* Rasnitsyn, 1966

  5. Sheath 2.2 mm long, 0.36× as long as forewing. Ovipositor narrow, possibly needle-like. ········································································ *E. karatavica* (Rasnitsyn, 1965)

  - Sheath 1.4–1.9 mm long. Ovipositor flat, not needle-like. ···································· 6

  6. Flagellomere 1 shorter than half the head width. Sheath 0.38× as long as forewing. ··············································· *E. pinicola* (Rasnitsyn in Krassilov et Rasnitsyn, 1982)

  - Flagellomere 1 longer than half the head width. Sheath 0.33× as long as forewing ············································································································· *E. eucalla* sp. nov.

Note. *E.? punctata* Rasnitsyn, and *E.? sibirica* Rasnitsyn, 1969 are not included in the key because of their insufficient knowledge.

**Figure 1:**
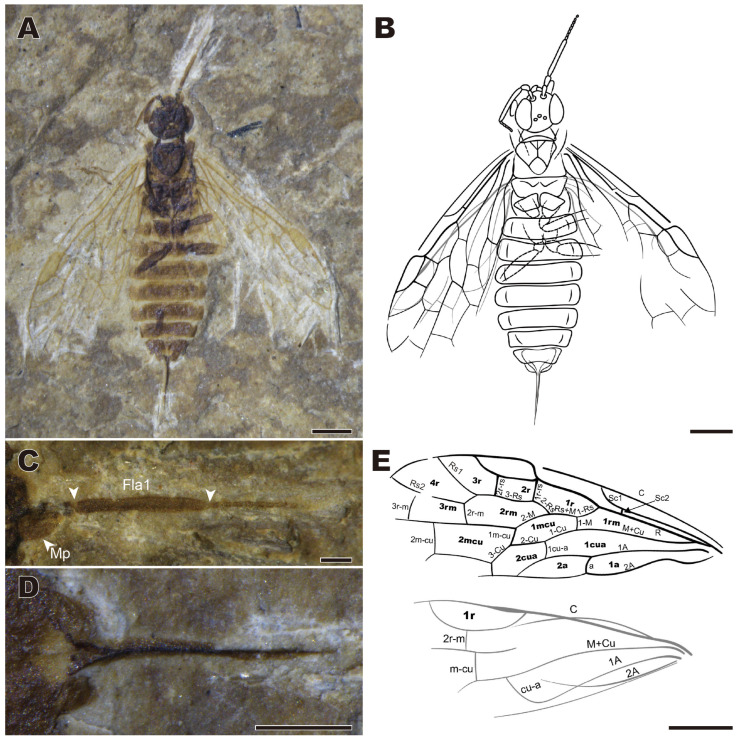
*Enneoxyela aculeata* sp. nov., holotype. CNU-HYM-LB2024101. (**A**) Dorsal view. (**B**) Line drawing from dorsal view. (**C**) Right antenna. (**D**) Ovipositor. (**E**) Venation of forewing and hind wing. Abbreviation: Fla1: flagellomere 1; Mp: maxillary palp; arrows in (**C**): boundary of Fla 1. Scale bars: (**A**,**B**,**E**): 1 mm; (**C**,**D**): 0.5 mm.

***Enneoxyela aculeata* sp. nov.** (Figure 1)

urn:lsid:zoobank.org:act:48297FE4-7DBE-4349-A343-39A17285528C

Holotype. CNU-HYM-LB2024101; female in dorsoventral position with nearly complete body and wings.

Etymology. The species name is derived from *aculeatus* (Latin), which means acicular and refers to the shape of the ovipositor.

Diagnosis. Body length without ovipositor approximately 7 mm, forewing ca. 5.5 mm long. Flagellomere 1 about as long as the head width. Forewing with R straight before Rs origin, 1-Rs almost twice as long as 1-M, 2-Rs near the middle of cell 1mcu (almost at 0.4 of its length), 2r-rs distal from the middle of pterostigma, whole pterostigma weakly sclerotized, cell 2a 3× as long as wide; ovipositor flat, short, with sheath ca. 0.25× as long as forewing.

Description. Holotype (female). Head, antenna, thorax, and femora dark brown, abdomen lighter brown, tibiae and tarsi apparently pale (not visible). Body length 6.69 mm and forewing 5.63 mm (Figure 1A,B).

Head rounded, 1.14 mm in width and 0.91 mm in length. Compound eyes large and bulge laterally. Antennae with scape thinner than pedicel and about 1.5× as long as pedicel, flagellomere 1 about as long as head width (Figure 1C). Maxillary palp with palpomere 3 enlarged. Ocelli in a low triangle.

Thorax with pronotum smooth, shortened mesally; mesonotum almost as wide as head, notauli short, V-shaped; metanotum with cenchri slightly wider than long.

Forewing with pterostigma weakly sclerotized, length 3× width; costal area broad, Sc parallel to C, with two branches, separated before 1-Rs; posterior branch (Sc_2_) very short, perpendicular to R; anterior branch (Sc_1_) much longer than Sc_2_, connected to C at level of Rs base; R slightly thickened and curved at Rs base; 1-Rs nearly as long as Rs+M and 2-Rs, nearly 2× as long as 1-M; 2-Rs near middle (almost at 0.4) of cell 1mcu; 1r-rs almost as long as 2r-rs, shorter than 3-Rs; 2r-rs distal of middle of pterostigma; M+Cu slightly curved; 1m-cu slightly shorter than 3-Cu; cell 1mcu slender, nearly 2.5× as long as wide; cell 2a nearly 3× as long as wide. Hind wing with 2r-m distad to m-cu; cell 1r more than 3.5× as long as wide (Figure 1E).

Legs incompletely preserved; femora slender, much longer than wide.

Abdomen with nine segments well preserved: ovipositor short, flat, sheath 1.4 mm long, tapering to an acute apex only distally (Figure 1D).

Male unknown.

Remarks. The new species is attributed to the genus *Enneoxyela* based on the combination of R straight before Rs base, Rs+M about twice as long as 1-M, and the straight ovipositor exserting for less than half of the forewing length. The new species differs from other congeners by the flagellomere 1 as long as the head width(much shorter than that in other species); from *E. karatavica* (Rasnitsyn, 1965), *E. compressicauda* Rasnitsyn, 1966, and *E. crassicauda* Rasnitsyn, 1966 by the shorter ovipositor sheath (longer than 2 mm in them). More distinguishing information can be found in the key above.

**Figure 2:**
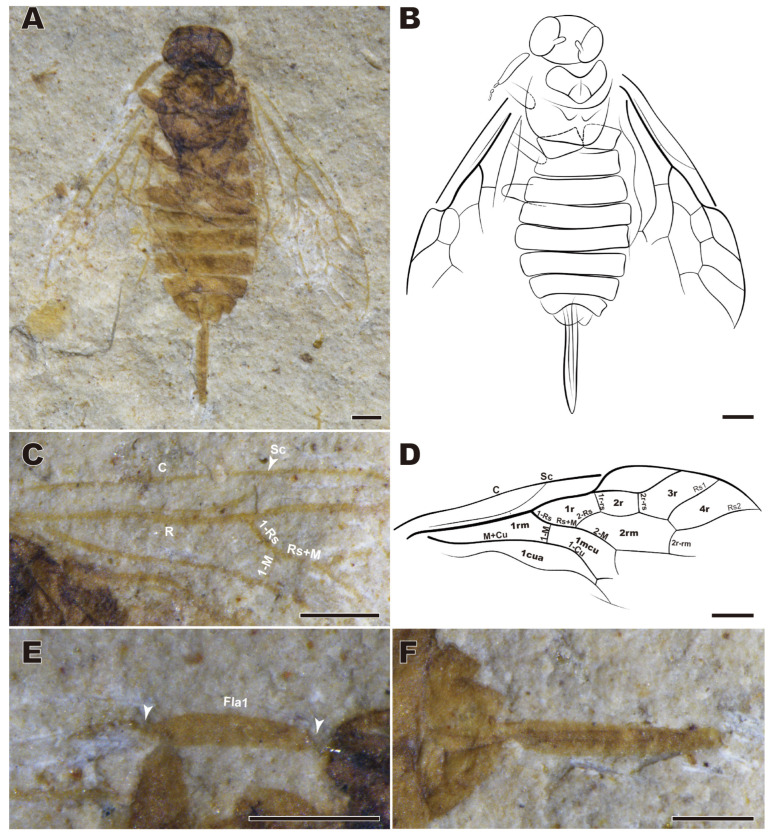
*Enneoxyela eucalla* sp. nov., holotype female. CNU-HYM-LB2024105. (**A**) Dorsal view. (**B**) Line drawing from dorsal view. (**C**) Part of the forewings. (**D**) Venation of forewing. (**E**) Left antenna. (**F**) Ovipositor. Abbreviation: Fla1: flagellomere 1; arrows in (**E**): boundary of Fla 1. Scale bars: 0.5 mm.

**Figure 3:**
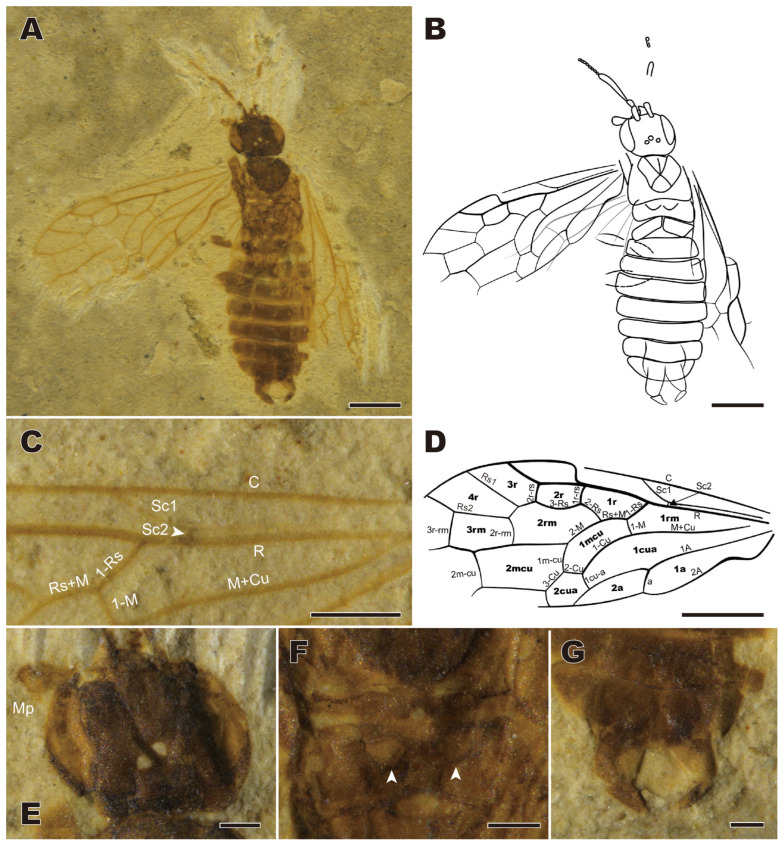
*Enneoxyela eucalla* sp. nov., paratype male. CNU-HYM-LB2024103. (**A**) Dorsal view. (**B**) Line drawing from dorsal view. (**C**) Part of the forewings. (**D**) Venation of forewing. (**E**) Head. (**F**) Metanotum. (**G**) male genitalia. Abbreviation and marks: Mp: maxillary palp; white arrows: cenchrus. Scale bars: (**A**–**D**): 1 mm; (**E**–**G**): 0.5 mm.

***Enneoxyela eucalla* sp. nov.** (Figure 2 and Figure 3)

urn:lsid:zoobank.org:act:600D1B12-C684-4348-B327-F175426D1F32

Holotype. CNU-HYM-LB2024105; female in dorsoventral position, with well-preserved body and incomplete wings (hind ones and posterodistal parts of forewings are insufficiently visible) (Figure 2).

Etymology. The species name is derived from *eucallos* (Greek), meaning beautiful, indicating the good preservation.

Diagnosis. Body length 5–6 mm, forewing length 4.3–4.5 mm. Flagellomere 1 ca. 0.6–0.7× as long as head wide. Ovipositor flat, straight, sheath 1.4 mm long, 0.35× as long as forewing.

Description. Female holotype. Head, thorax, and femora dark brown, antenna and abdomen light brown, tibiae and tarsi apparently pale. Body length 4.9 mm, forewing length 4.35 mm (Figure 2A,B).

Head oblate, 0.70 mm long and 1.15 mm wide. Compound eyes are large and bulging. Antennae with flagellomere 1 distinctly shorter than head width, remaining flagellomeres combined slightly longer than half of flagellomere 1; scape and pedicel thick (Figure 2E).

Thoraxinsufficiently preserved.

Forewing with pterostigma sclerotized marginally; Sc approaching R, Sc_2_ hardly visible; 1-Rs slightly longer than 1-M, 2-Rs at mid-length of cell 1mcu; 1-Cu strongly bent (Figure 2C,D).

The abdomen nine segments and well preserved. Ovipositor straight, flat, sheath 1.4 mm long, parallel-sided, converging only at apex (Figure 2F).

Male paratype CNU-HYM-LB2024103 in dorsoventral position, completely preserved except for tibiae and tarsi invisible (probably pale in color) and right wings and left hind wing crumpled and difficult to interpret (Figure 3).

Body including femora and antennomeres 1–3 dark brown, antennal thread and wing venation paler. Body length excluding antennae 5.73 mm, forewing length 4.43 mm (Figure 3A,B).

Head oblate, 0.80 mm long and 1.05 mm wide. Eyes large, almost as long as the head, not bulging. Antennae incomplete with flagellomere 1 0.7× as long as head wide, thicker than other flagellomeres; scape and pedicel short and thick, with length ratio 1.4:1. Maxillary palpomere 3 much enlarged. Ocelli in a low triangle (Figure 3E).

Thorax width nearly equal to that of the head. Notauli short. Cenchri slightly wider than long (Figure 3F).

Forewing with pterostigma slightly sclerotized, ca. 3× as long as wide. The coastal area comparably narrow. Sc weak, running close to R; Sc_2_ very short, perpendicular to R; R thickened distally. 1-Rs 1.5× as long as 1-M; 2-Rs near basal third of cell 1mcu; 1r-rs as long as 2r-rs, shorter than 3-Rs; 2r-rs distal from the middle of pterostigma; M+Cu slightly curved; 1-Cu strongly bent and elongated, cell 1mcu slender with an obvious bent; 2-Cu short, almost as long as 1cu-a; 1m-cu nearly as long as 3-Cu; cell 2a more than 3× as long as wide (Figure 3C,D).

Legs incompletely preserved, with femora slender.

Abdomen with nine segments well preserved; genitalia twisted for 180°; gonostylus elongate, with distinct gonomacula (Figure 3G).

Remarks. The new species is attributed to the genus *Enneoxyela* based on R straight before the origin of Rs; 2r-rs near pterostigma midlength; Rs1 apex closer to Rs2 than to pterostigma. The female sex of the new species can be distinguished from other congeners in that the saw sheath is only 1.4 mm, from *E. pinicola* in that flagellomere 1 is distinctly longer than half of the head width. The male of the new species is the first recorded for *Enneoxyela* and so cannot be compared with congeners.

**Figure 4:**
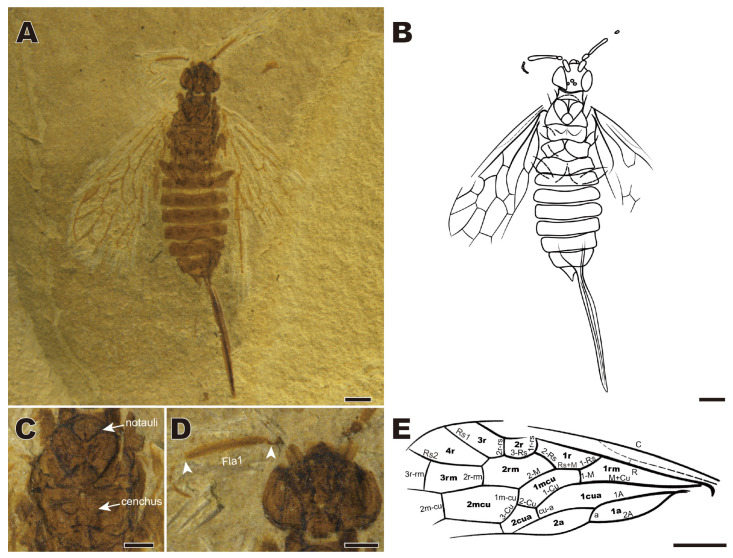
*Hemixyela elongata* gen. et sp. nov., holotype. CNU-HYM-LB2024104. (**A**) Dorsal view. (**B**) Line drawing from dorsal view. (**C**) Thorax. (**D**) Head and left antenna. (**E**) Venation of forewing. Abbreviation: Fla1: flagellomere 1; arrows in (**D**): boundary of Fla 1. Scale bars: 1 mm.

***Hemixyela* gen. nov.** (Figure 4)

urn:lsid:zoobank.org:act:479608F2-4AE6-42EA-ACC3-28A94A0CF5DD

Type species: *Hemixyela elongata* sp. nov.; Early Cretaceous of NE China.

Etymology. Genus name combines *hemi-* (Greek) meaning half and genus name *Xyela*, and refers to the position of the forewing 2r-rs at the middle of the pterostigma.

Diagnosis. Sc branches ending at C distal of, and at R distinctly basal of, Rs base. R straight before the Rs base. Rs+M about twice as long as 1-M. 2r-rs near midlength of pterostigma. 1m-cu and 2-Cu about as long as 1-M. Ovipositor straight, exserting for more than half of the forewing length.

Species included. Type species only.

Remarks. *Hemixyela* gen. nov. is attributed to tribe Xyelini by 1-Rs longer than 1-M; 3-Cu longer than 1m-cu. It differs from all except *Spathoxyela* by a long ovipositor, from *Spathoxyela* by the submedial position of 2r-rs at the pterostigma and long Rs+M.

***Hemixyela elongata* gen. et. sp. nov.** (Figure 4)

urn:lsid:zoobank.org:act:EB46252E-B06F-4D04-BB3C-1277C1102898

Holotype. CNU-HYM-LB2024104; female in dorsoventral position with well-preserved body, left forewing, and incomplete other wing and legs.

Etymology. The species name is derived from *elongatus* (Latin), meaning long, referring to the long ovipositor.

Diagnosis. As for the genus.

Description. Color brown except paler antennal thread, wing venation, tibiae, and tarsi. Body length 8.32 mm excluding antennae and ovipositor; forewing length 6.34 mm (Figure 4A,B).

Head rounded, 1.49 mm wide and 1.05 mm long. Compound eyes huge, occupying 1/3 of the width of the head from a dorsal view. Antennae incomplete, with scape thicker than other segments and about twice as long as pedicel, flagellomere 1 0.75× as long as head wide (Figure 4D).

Thorax with pronotum almost 0.6× as wide as head; mesonotum with notauli and median line well developed; cenchri longer than wide (Figure 4C).

Forewing with pterostigma lanceolate, with ratio length: width = 4:1; costal area comparably narrow; Sc hardly visible (marked with dotted lines in Figure 4E), R thickened at base; 1-Rs ca. 1.5× as long as 1-M; 2-Rs near basal 1/3 of cell 1mcu; 1r-rs slightly shorter than 2r-rs, shorter than 3-Rs; 2r-rs near middle of pterostigma; M+Cu slightly curved and thickened; 1-Cu strongly bent and elongated, thus cell 1mcu slender with an obvious bent; 2-Cu short but longer than 1cu-a; 1m-cu shorter than 3-Cu; length of cell 2a more than 3× width (Figure 4E).

Legs with slender femora, hind tibia long and slender, as long as wing cells 3r and 4r combined, hind tarsus particularly slender, longer than tibia.

Abdomen with nine segments well preserved, with ovipositor long, straight, sheath 4.8 mm long, tapering to apex in its distal third, 0.7× as long as forewing and 3× as long as valvifer 2 (basal plate of ovipositor).

Male unknown.

**Figure 5:**
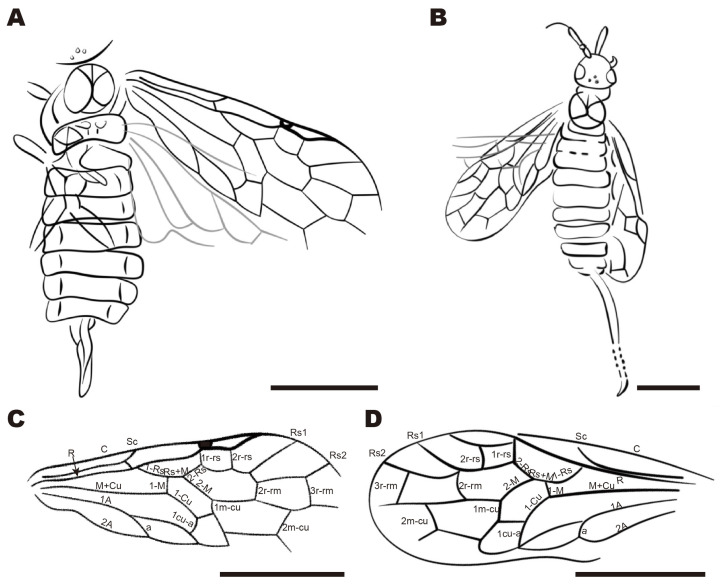
(**A**,**C**) *Tugnuxyela tugnuica* Rasnitsyn, 1983, holotype, modified from Rasnitsyn, 1983, (**A),** modified line drawing of body (**C**) Venation of forewing. (**B**,**D**) *Junfengixyela cenozoica* Zhang, 1989, holotype, modified from Zhang et al., 1989, (**B**) modified line drawing from dorsal view. (**D**) Venation of forewing. Scale bars: 1 mm. [17,18].

**Genus *Tugnuxyela* gen. nov.** (Figure 5A,C)

urn:lsid:zoobank.org:act:11421E8D-C1E5-4108-A919-D8AD29526ED0

Type species. *Eoxyela tugnuica* Rasnitsyn, 1983

Etymology. The genus name combines the names of the type species (partially) and genus *Xyela*. Gender feminine.

Diagnosis. Rs+M about as long as 1-M. 2r-rs near midlength of pterostigma. Apex of Rs1 much closer to the pterostigma than to Rs2. Sc branches ending at C and R at about the same level before the Rs base. Ovipositor short, downcurved.

Species included. Type species from the Upper Lower Cretaceous of Transbaikalian in Russia [17].

Remarks. The new genus belongs to a group of genera with R straight before Rs base: it differs from other genera in that group in having Sc with two subequal branches ending at C and R distinctly before Rs base (like in Pleroneura that differs by R sinuate before Rs base).

**Genus *Junfengixyela* gen. nov.** (Figure 5B,D)

urn:lsid:zoobank.org:act:9BABEC09-E033-42B1-BCAA-3B0B46A57C5B

Type species. *Xyela cenozoica* Zhang, 1989

Etymology. The genus name combines the first name of the author of the type species (Jun-feng Zhang) and the name of the genus *Xyela*. Gender feminine.

Diagnosis. R straight before the Rs base. Rs+M about twice as long as 1-M. 2r-rs near midlength of pterostigma. Apex of Rs1 slightly closer to pterostigma than to Rs2. Sc branches ending at C distal of, and at R distinctly basal of, Rs base. Ovipositor long, slightly upcurved, tapering gradually toward an acute apex.

Species included. Type species from the middle Miocene of Shandong in China [18].

Remarks. The new genus belongs to a group of genera with R straight before Rs base: it differs from other genera in that group in having an ovipositor long, flat, slightly upcurved, and tapering gradually toward an acute apex.

## 4. Discussion

Huangbanjigou is one of the well-known Mesozoic fossil localities in China and a part of the Jehol Biota. Early birds, reptiles, angiosperms, and other taxa have been reported here [19,20]. Similarly, abundant fossil records of xyelids have been discovered in this area; however, all previous records belonged to the subfamily Macxyelinae [21,22]. Here we report a valuable assemblage of Xyelinae fossils from this locality, the first one for the Cretaceous of China, which enriches the diversity of Xyelidae in general. These fossil records all belong to the tribe Xyelini and exhibit high species diversity, which not only fills existing gaps in the fossil record of Xyelinae but also provides conditions for the revision and reorganization of Mesozoic taxa within Xyelini.

The male paratype of *Enneoxyela eucalla* sp. nov. is the first record of a male Xyelini in the Mesozoic. Of special importance is the observation of its genitalia twisted for 180° (strophandry), a feature characteristic of living Xyelini and otherwise uncommon in Hymenoptera (also found in some Tenthredinoidea excluding Blasticotomidae). This observation supports attribution of the Mesozoic genera of Xyelini to that tribe. Beyond its diagnostic value, the presence of strophandry in this ancient specimen suggests that the associated functional advantages probably arose early in the group’s evolution. Functionally, this strophandrous condition provides a direct mechanical advantage during copulation; it inherently positions the genitalia in the orientation for mating, thereby eliminating the need for the abdominal twisting required in orthandrous species [23]. This strophandrous mating strategy is thought to conserve time and energy compared to the orthandrous condition, thereby reducing the risk of predation [23].

During the examination of the new species, we reviewed the entire system of the tribe. We have refined diagnostics of the genera involved, which resulted in the description of new genera and changed the generic attribution of some species. We summarized the changes proposed in the original keys to genera of Xyelini and to species of *Enneoxyela*, the genus richest in species number among the Mesozoic Xyelinae. A total of three new genera are established: *Hemixyela* gen. nov. for a new species from the Lower Cretaceous Yixian Formation in NE China, *Tugnuxyela* gen. nov. for the species previously described from the upper Lower Jurassic Ichetuy Formation in Eastern Siberia, and *Junfengixyela* gen. nov. for the previously described Miocene species from the Shanwang Formation in NE China. Two new species from the Yixian Formation are described in the genus *Enneoxyela*. In addition, we provide an updated catalog of fossil species of Xyelini (Table 1), a total of nine genera and 23 species. Mesozoic representatives of this group have been primarily documented from three of the following regions in the Northern Hemisphere: Russia, Kazakhstan, and China. Later occurrences are recorded in Cenozoic strata from Germany and the United States, while extant distributions are confined to the Holarctic and Oriental regions [24] (Table 1). It is tempting to hypothesize that the group originated in the Palaearctic region and subsequently dispersed into the Oriental and Nearctic regions. This may well be the case. However, the limitation of the Mesozoic distribution of Xyelini to Asia might result, at least in part, because of insufficient knowledge of the extra-Asiatic Jurassic and Early Cretaceous insect fossils record. It is a matter of future research to check out if the current strictly Asiatic distribution of Xyelini during the Jurassic and Early Cretaceous was real or only an artifact of uneven research efforts.

Results of the present work suggest that our knowledge of the past diversity of Xyelini is insufficient yet. We expect that the keys provided will be of help in the identification of Xyelini fossils to be found in Mesozoic and Cenozoic deposits, which will further fill the gaps in our knowledge and enrich our understanding of the past taxonomical and morphological diversity of that important group of basal Hymenoptera. This might open the way to more in-depth studies of phylogeny, biology, and other aspects of paleobiology of Xyelini.

## Figures and Tables

**Table 1:** Fossil species of Xyelini Newman 1834.

Tribe	Genus	Subgenus	Species	Geological Age	Location	Former Name
Xyelini Newman 1834	*Enneoxyela* Rasnitsyn, 1966		*Enneoxyela aculeata* sp. nov.	Cretaceous	China	
			*Enneoxyela atra* Rasnitsyn, 1966	Jurassic	Kazakhstan	*Eoxyela atra* Rasnitsyn 1966
			*Enneoxyela compressicauda* Rasnitsyn, 1966	Jurassic	Kazakhstan	
			*Enneoxyela crassicauda* Rasnitsyn, 1966	Jurassic	Kazakhstan	
			*Enneoxyela eucalla* sp. nov.	Cretaceous	China	
			*Enneoxyela karatavica* Rasnitsyn, 1965	Jurassic	Kazakhstan	
			*Enneoxyela pinicola* Rasnitsyn, 1982	Cretaceous	Russian	*Spathoxyela pinicola* Rasnitsyn 1982
			*Enneoxyela punctata* Rasnitsyn, 1965	Jurassic	Kazakhstan	*Eoxyela punctata* Rasnitsyn 1965
			*Enneoxyela sibirica* Rasnitsyn, 1969	Cretaceous	Russian	*Eoxyela sibirica* Rasnitsyn 1969
	*Eoxyela* Rasnitsyn, 1965		*Eoxyela scoliura* Rasnitsyn, 1965	Jurassic	Kazakhstan	
	*Hemixyela* gen. nov.		*Hemixyela elongata* gen. et sp. nov.	Cretaceous	China	
	*Junfengixyela* gen. nov.		*Junfengixyela cenozoica* Zhang, 1989	Miocene	China	*Enneoxyela cenozoica* Zhang 1989
	*Spathoxyela* Rasnitsyn, 1969		*Spathoxyela fossilis* Rasnitsyn, 1965	Cretaceous	Russian	
	*Tugnuxyela* gen. nov.		*Tugnuxyela tugnuica* Rasnitsyn, 1983	Jurassic	Russian	*Eoxyela tugnuica* Rasnitsyn 1983
	*Xyela* Dalman, 1819	*Xyela (Mesoxyela)* Rasnitsyn, 1965	*Xyela (Mesoxyela) mesozoica* Rasnitsyn 1965	Cretaceous	Russian	
		*Xyela (Pinicolites)* Meunier 1920	*Xyela (Pinicolites) graciosa* Meunier 1920	Oligocene	Germany	
		*Xyela (Xyela)* Dalman, 1819	*Xyela (Xyela) angustipennis* Statz, 1936	Miocene	Germany	
			*Xyela (Xyela) florissantensis* Rasnitsyn, 1995	Eocene	the United States	
			*Xyela (Xyela) latipennis* Statz, 1936	Oligocene	Germany	
			*Xyela (Xyela) magna* Statz 1936	Oligocene	Germany	
			*Xyela (Xyela) micrura* Rasnitsyn, 1995	Oligocene	Germany	
	*Xyelisca* Rasnitsyn, 1969		*Xyelisca leptopoda* Rasnitsyn, 1969	Jurassic	Russian	
	*Yanoxyela* Ren, Lu, Guo, and Ji, 1995		*Yanoxyela hongi* Ren, Lu, Guo, and Ji, 1995	Jurassic	China	

## Data Availability

The original contributions presented in this study are included in the article. Further inquiries can be directed to the corresponding author.
